# Synergistic potential of Ivermectin and doxorubicin in oral squamous cell carcinoma: an in vitro investigation

**DOI:** 10.1186/s40360-025-01053-4

**Published:** 2025-12-12

**Authors:** Rana Tantawy, Shereen Nader Raafat, Ayman El-Gawish, Dalia Ghalwash

**Affiliations:** 1 Oral Medicine and Periodontology, Faculty of Dentistry, The British University in Egypt, El Sherouk City, Egypt; 2 Department of Pharmacology, Faculty of Dentistry, The British University in Egypt, El Sherouk City, Egypt; 3 Dental Science Research Group, Health Research Centre of Excellence, The British University in Egypt (BUE), El Sherouk City, Egypt

**Keywords:** Doxorubicin, Ivermectin, Oral squamous cell carcinoma, Apoptosis, Oxidative stress, Combination therapy

## Abstract

**Background:**

Doxorubicin (DOX) is widely used in cancer therapy, but its role in oral squamous cell carcinoma (OSCC) is limited by resistance and dose-related toxicities. Ivermectin (IVM), an antiparasitic agent with emerging anticancer properties, may enhance DOX efficacy. This study evaluated the effects of IVM alone and in combination with DOX on OSCC cell lines.

**Methods:**

In vitro assays, including MTT viability, apoptosis (Annexin V/PI), cell cycle analysis, RT-qPCR of apoptotic, proliferative, and inflammatory markers, and oxidative stress assays, were performed on HN9 and HEp-2 OSCC cell lines, with OEC as control.

**Results:**

IVM reduced cancer cell viability in a dose-dependent manner and demonstrated a favorable selectivity profile compared to normal cells. Combination treatment with DOX and IVM significantly enhanced cytotoxicity (CI = 0.368, synergistic), induced S-phase cell cycle arrest, and increased apoptosis through upregulation of BAX, Caspase-3, and P53, alongside downregulation of BCL2. The combination also suppressed Ki-67 and IL-6 expression and markedly increased oxidative stress, indicating mitochondrial dysfunction.

**Conclusion:**

IVM exhibits anticancer activity in OSCC cells and synergistically augments the efficacy of DOX. These findings support the potential of DOX + IVM combination therapy as a novel strategy for OSCC, warranting further validation in in vivo and clinical studies.

**Supplementary Information:**

The online version contains supplementary material available at 10.1186/s40360-025-01053-4.

## Introduction

Head and neck squamous cell carcinomas (HNSCC) rank as the seventh most prevalent cancer globally, with a fatal outcome in half of the diagnosed cases [1]. Oral squamous cell carcinoma (OSCC), which accounts for approximately 90% of HNSCCs [2], is the most common malignancy of the oral cavity [3]. Tobacco smoking, alcohol consumption, betel quid chewing, areca nut usage, human papillomavirus (HPV) infection, and genetic predisposition are identified as potential risk factors for OSCC [4]. In 2022, the estimated global incidence of oral cancer was 389,846 new cases, with 188,438 associated deaths [5]. The treatment modalities for OSCC mainly include surgery, chemotherapy, radiotherapy, or combinations of these approaches [6].

Despite the implementation of advanced therapeutic strategies, the current five-year survival rate for OSCC remains approximately 50–60% [7–9]. Platinum-based chemotherapeutics and anthracyclines, including doxorubicin (DOX), are the most versatile cytotoxic agents in cancer therapy. DOX is a widely used chemotherapeutic agent as a first-line therapy for various malignancies, such as carcinomas, breast cancer, sarcomas, and hematological malignancies [10, 11]. Despite its high therapeutic efficacy, the clinical application of DOX is restricted by the development of chemoresistance and its narrow therapeutic index, which is attributed to concentration-dependent toxicities, such as cardiotoxicity and myelosuppression [12, 13]. These challenges reduce its therapeutic efficacy and contribute to treatment failure and tumor recurrence in OSCC [14], highlighting the pressing need to find novel therapeutic strategies capable of overcoming drug resistance, enhancing anticancer activity, and minimizing associated toxicities. However, developing novel anti-cancer treatment is a costly and time-consuming process that requires pre-clinical in vitro and in vivo assays, followed by clinical trials [15].

In this context, drug repositioning or repurposing offers a promising approach for chemotherapy research. Drug repositioning refers to expanding the therapeutic applications of clinically approved drugs beyond the scope of their original therapeutic use [16]. Ivermectin (IVM), a macrolide antiparasitic drug, is currently investigated for repurposing as a potential anti-cancer therapeutic agent. Studies have reported its anticancer effectiveness against a range of malignancies through modulating multiple oncogenic pathways, such as regulating tumor microenvironment, inhibition of angiogenesis and metastasis, as well as induction of oxidative stress, and subsequently mitochondrial dysfunction [17].

Combination therapy has recently emerged as an effective strategy in cancer management, showing superior efficacy over monotherapy by simultaneously targeting multiple oncogenic pathways, improving patient outcomes, and overcoming drug resistance, particularly in oral cancer [15, 18]. IVM has been reported to enhance the effectiveness of standard chemotherapeutic agents by acting as a chemosensitizer by blocking drug efflux and reversing drug resistance [19]. For instance, IVM enhanced the cytotoxic effects of cisplatin and 5-fluorouracil in esophageal squamous cell carcinoma cell lines by triggering both the extrinsic and intrinsic apoptotic pathways [20]. In hepatocellular carcinoma cells, IVM significantly potentiated the therapeutic efficacy of sorafenib by inhibition of mTOR/STAT3 pathway [21]. Additionally, IVM augmented the anti-cancer efficacy of cisplatin in ovarian cancer, both in vitro and in vivo models, by inhibiting the Akt/mTOR pathway, thus blocking downstream signaling required for cancer cell proliferation and survival [22]. Therefore, combining IVM with an established chemotherapeutic agent, such as DOX, could enhance the sensitivity of OSCC cells to DOX and may contribute to reducing the dose, toxicity, and resistance associated with it.

Based on thorough search in the literature, IVM anti-cancer efficacy has not yet been investigated in the context of oral cancer. Therefore, the aim of this study was to elucidate the anti-cancer potential of IVM in oral cancer cell lines and to evaluate the effects of combining IVM with DOX on oral cancer cell lines. In the present study, an MTT assay was utilized to assess cell viability, an annexin V/propidium iodide (PI) apoptosis assay was performed to evaluate apoptosis, and flow cytometry was used to analyze cell cycle progression. This study also examined the expression of apoptotic, anti-apoptotic, proliferative, and pro-inflammatory markers using RT-qPCR. Finally, oxidative stress was evaluated by measuring the concentration of reduced glutathione (GSH) to clarify the molecular mechanisms underlying the anti-cancer effects of IVM and the DOX + IVM synergistic interaction.

## Materials and methods

### Materials

The Human Tongue Squamous Cell Carcinoma (HN9) cell line and the normal oral epithelial cell line (OEC) were purchased from the Nanotechnology Centre (NRC), The British University in Egypt (Cairo, Egypt). The HEp-2 cell line, representing the Human Laryngeal Squamous Cell Carcinoma, was procured from Nawah Scientific (Cairo, Egypt). Ivermectin was purchased in powder form from Sigma, while Doxorubicin was bought from Khandelwal Laboratories Pvt Ltd (India). Cell culture media, including Dulbecco’s Modified Eagle’s Medium (DMEM) and fetal bovine serum (FBS), were obtained from Gibco^®^, Thermo Fisher Scientific (USA). The antibiotic-antimycotic mixture was obtained from Lonza^®^ (USA) and contained 100 U/mL of penicillin, 0.1 U/mL of streptomycin, and 0.25 µg/mL of amphotericin B.

### Cell culture

OEC, HN9, and HEp-2 cells were cultured in a DMEM high-glucose medium supplemented with 10% FBS and 1% antibiotic/antimycotic mix till 80% confluency at 37 °C and 5% CO₂ in a humidified incubator. Cell culture procedures were carried out within a Class II laminar flow hood, and incubations were performed at 37 °C in an incubator.

### Cell viability assay (MTT assay)

The cytotoxicity of DOX and IVM on OEC, HN9, and HEp-2 cancer cells was assessed using the MTT assay (3-[4,5-dimethylthiazol-2-yl] -2,5-diphenyl tetrazolium bromide). Cells were seeded (10 × 10^3^ cells / well) in 0.2 mL of medium in a 96-well plate and incubated for 24 h. Cells were treated with serial dilutions of DOX (200 µM – 1.56 µM) and IVM formulation (30 µM − 0.23 µM) [23] on the following day. Cell culture medium was exchanged with 100 µL of 5 mg/mL MTT solution in each well after 48 h. The cells were incubated for 4 h, after which the supernatant was discarded, and 100 µL/well of DMSO was added to dissolve the formazan crystals [24]. Absorbance was measured utilizing a microplate reader (Thermo Scientific Multiscan GO, USA) at 570 nm. The 50% and 20% inhibitory concentrations (IC₅₀ and IC₂₀) of the drugs were then calculated by GraphPad Prism software utilizing non-linear regression analysis.

$$\mathrm{Combination}\:\mathrm{index}\left(\mathrm{CI}\right)=\frac{\mathrm{d}1}{\mathrm{D}1}+\frac{\mathrm{d}2}{\mathrm{D}2}$$

where d1 and d2 are the concentrations of IVM and DOX used in combination to reach 50% cell viability, and D1 and D2 are the concentrations of the drug causing 50% cell viability when used alone. The effect of the combination is reported to be synergistic if the CI is < 0.8, additive if the CI ranges from 0.8 to 1.2, and antagonistic if the CI >1.2 [25]. The determined drugs concentrations and combination were compared using cancer and the normal cell lines (OEC) to explore the combination selectivity. An inverted microscope was used to visualize the cells at 10x, 20x, and 4x magnifications.

### Apoptosis assay

For the apoptosis assay, 4 × 10⁶ cells were cultured in T-75 flasks and permitted to adhere overnight. Cells were exposed to DOX (IC_20_), IVM (1.79 µM), and their combination on the subsequent day and left for 48 h, while fresh medium was kept as the control group. The cells that received treatment were exposed to trypsin, then washed twice with PBS and centrifuged at 280 xg for 5 min to harvest the cells. The cell pellet was redispersed in PBS and maintained on ice until flow cytometry analysis. 100 µL of cell suspension from each sample was incubated in the dark for 15 min with 5 µL of Annexin V-FITC and 1 µL of PI stock solution (100 µg/mL). Subsequently, 400 µL of 1× Annexin binding buffer was applied to each sample, which was then analyzed using a CytoFlex flow cytometer (Beckman Coulter, CA, USA) following the manufacturer’s instructions. A minimum of 10,000 events was recorded for each sample. The data obtained were interpreted with CytExpert software (Beckman Coulter, CA, USA).

### Cell cycle analysis

After cell treatment, as previously mentioned, the cell pellets were resuspended in 2 mL cold ethanol (60%) and kept for 1 h at 4 °C, then washed twice with PBS for cell fixation. Cells were resuspended in 1 mL of a nucleic acid staining solution (10 µg/mL PI and 50 µg/mL RNase A in PBS) and incubated in the dark at 37 °C for 20 min. CytExpert software (Beckman Coulter, CA, USA) was employed to determine cell cycle distribution; at least 10,000 events were captured.

### Apoptosis, proliferation, and inflammation-related markers analysis

The expression of apoptosis-related markers (BAX, Caspase-3, and P53), anti-apoptotic marker (BCL2), cell proliferation marker (Ki-67), and pro-inflammatory marker (IL-6) was assessed using real-time quantitative PCR (RT-qPCR). Cells (7 × 10⁵) were cultured in triplicate T-25 flasks and exposed to DOX (IC_20_), IVM (1.79 µM), or their combination for 48 h. Control groups were cells seeded in a normal culture medium. Following cell harvesting, RNA extraction from the cell pellets was performed through the QIAGEN RNA extraction kit (QIAGEN, Hilden, Germany) according to the manufacturer’s guidelines. RevertAid First Strand cDNA Synthesis Kit (Thermo Scientific, MA, USA) was utilized for Complementary DNA (cDNA) synthesis and reverse transcription. The resulting cDNA was then amplified and quantified employing SYBR Green Supermix (Bio-Rad) on a Bio-Rad RT-qPCR system. The mRNA expression levels were normalized to GAPDH, which served as the reference housekeeping gene. The 2⁻ΔΔCt method was utilized for data analysis [24]. All samples were evaluated three times (*n* = 3), and the experiment was conducted in triplicate. Results are presented as fold changes relative to the control group. The sequence of the primers utilized is listed in Table 1.

**Table 1:** The sequence of primers utilized in the study

Gene	Forward primer	Reverse primer
BAX	GCCTCCTCTCCTACTTTG	CTCAGCCCATCTTCTTCC
BCL2	ACAGGAGCTATACTCCAGGACA	GATCATACCCGTCATGGGGATA
Caspase-3	CATGGAAGCGAATCAATGGACT	CTGTACCAGACCGAGATGTCA
P53	ACTTGTCGCTCTTGAAGCTAC	GATGCGGAGAATCTTTGGAACA
Ki-67	AGAAGAAGTGGTGCTTCGGAA	AGTTTGCGTGGCCTGTACTAA
IL-6	ACTCACCTCTTCAGAACGAATTG	CCATCTTTGGAAGGTTCAGGTTG
GAPDH	GGAGCGAGATCCCTCCAAAAT	GGCTGTTGTCATACTTCTCATGG

### Oxidative marker assay

To further elucidate the mechanism underlying the interaction between DOX and IVM, oxidative stress was assessed in HN9, and HEp-2 cell lines by measuring the concentration of reduced glutathione (GSH). The cells underwent repeated freeze-thaw processes multiple times until complete lysis of the cells was achieved. The supernatant was collected for further analysis using a commercial kit (Bio Diagnostic, Egypt). 5,5’-dithiobis (2-nitrobenzoic acid) (DNTB) is reduced by glutathione (GSH) within the sample, resulting in the formation of the yellow product, 5-thio-2-nitrobenzoic acid (TNB). Absorbance of the reduced chromogen was measured at 405 nm using a plate reader (BMG Labtech, FLUOstar Omega, Germany) [26].

### Statistical assessment

Each experiment was conducted at least three times, with three to six replicates per treatment and drug combination. Statistical analysis was performed with GraphPad Prism software version 6.0, utilizing one-way or two-way ANOVA, followed by post-hoc Tukey’s test. The obtained data are presented as mean ± standard deviation (SD). A p-value less than 0.05 is deemed statistically significant.

## Results

### Drug cytotoxicity assay

**Fig. 1:**
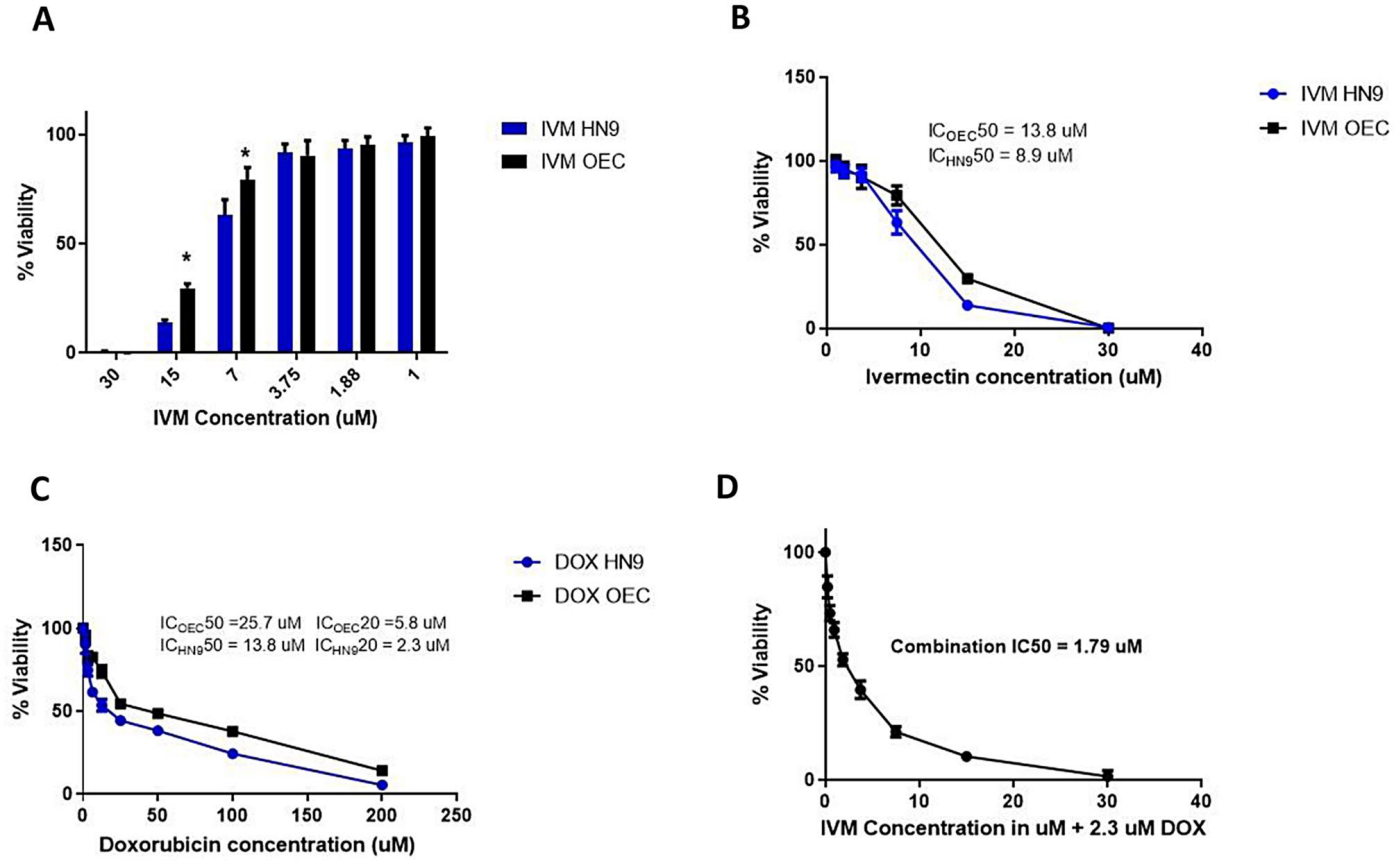
MTT assays performed after 48 h treatment with DOX and IVM drugs. **(A)** MTT assay showing the dose-dependent effects using serial dilutions of IVM on the cell viability in OEC and HN9 cell lines after 48 h of treatment. **(B)** MTT assay showing results of the effect of IVM on OEC and HN9 cell lines after 48 h. of treatment **(C)** MTT assay demonstrates the effects of DOX on the OEC and HN9 cell lines after 48 h of treatment. **(D)** MTT assay showing the effect of using serial dilutions of IVM combined with the IC_20_ value of DOX on HN9 cells after 48 h of treatment

To explore the cytotoxic effects of DOX and IVM on OEC and HN9 cell lines, an MTT assay was performed. Both cell lines were treated with serial dilutions of DOX (200 µM – 1.56 µM) and IVM (30 µM − 0.23 µM), resulting in a dose-dependent decrease in cell viability with both drugs. The results revealed that IVM exhibited a significant cytotoxic effect on both cell lines at concentrations of 30 and 15 µM. At 7.5 µM concentration, IVM showed significant cytotoxicity on HN9 cells compared to OEC. In contrast, IVM concentrations at or below 3.75 µM maintained high cell viability (> 90%), suggesting a safer profile for both cell lines (Fig. 1A).

For IVM, the IC_50_ was calculated to be 8.9 µM in HN9 cells and 13.8 µM in OEC cells (Fig. 1B). The IC_50_ value for DOX in HN9 cells was 13.8 µM and 25.7 µM in OEC cells, denoting a higher cytotoxic effect on HN9. The IC_20_ values for DOX were found to be 2.3 µM in HN9 cells and 5.8 µM in OEC cells (Fig. 1C).

To identify the optimal concentration of DOX and IVM for combination therapy, we initially tested the IC_50_ of both DOX and IVM, which resulted in significant toxicity to both normal and cancerous cells, resulting in nearly complete cell death. To identify the optimal concentration of IVM to be used in combination therapy, both cell lines were treated with serial dilutions of IVM with the IC_20_ value of DOX. The IVM concentration that caused 50% cell viability in combination with the IC_20_ of DOX was 1.79 µM (Fig. 1D).

Based on these findings, 1.79 µM IVM was used in subsequent assays, as it was safe when used alone and induced 50% (IC_**50**_) cell viability when combined with 2.3 µM DOX (IC_**20**_), thereby maintaining an adequate number of cells for subsequent molecular analysis. The CI determined using the aforementioned drug concentrations was 0.368 (< 1), indicating a synergistic effect. The mentioned drugs concentrations have proven to be relatively safe when tested on OEC normal cells, indicating drugs selectivity (Supplementary data A).

Upon microscopic investigation, the cytotoxic effect of the investigated drugs combination induced marked changes in cancer cells morphology, such as rounding, shrinkage, and detachment (Supplementary data B).

To ensure the effectiveness and validity of the obtained results, the HEp-2 cell line was used in subsequent experiments using the same drug concentrations.

### Apoptosis assay

**Fig. 2:**
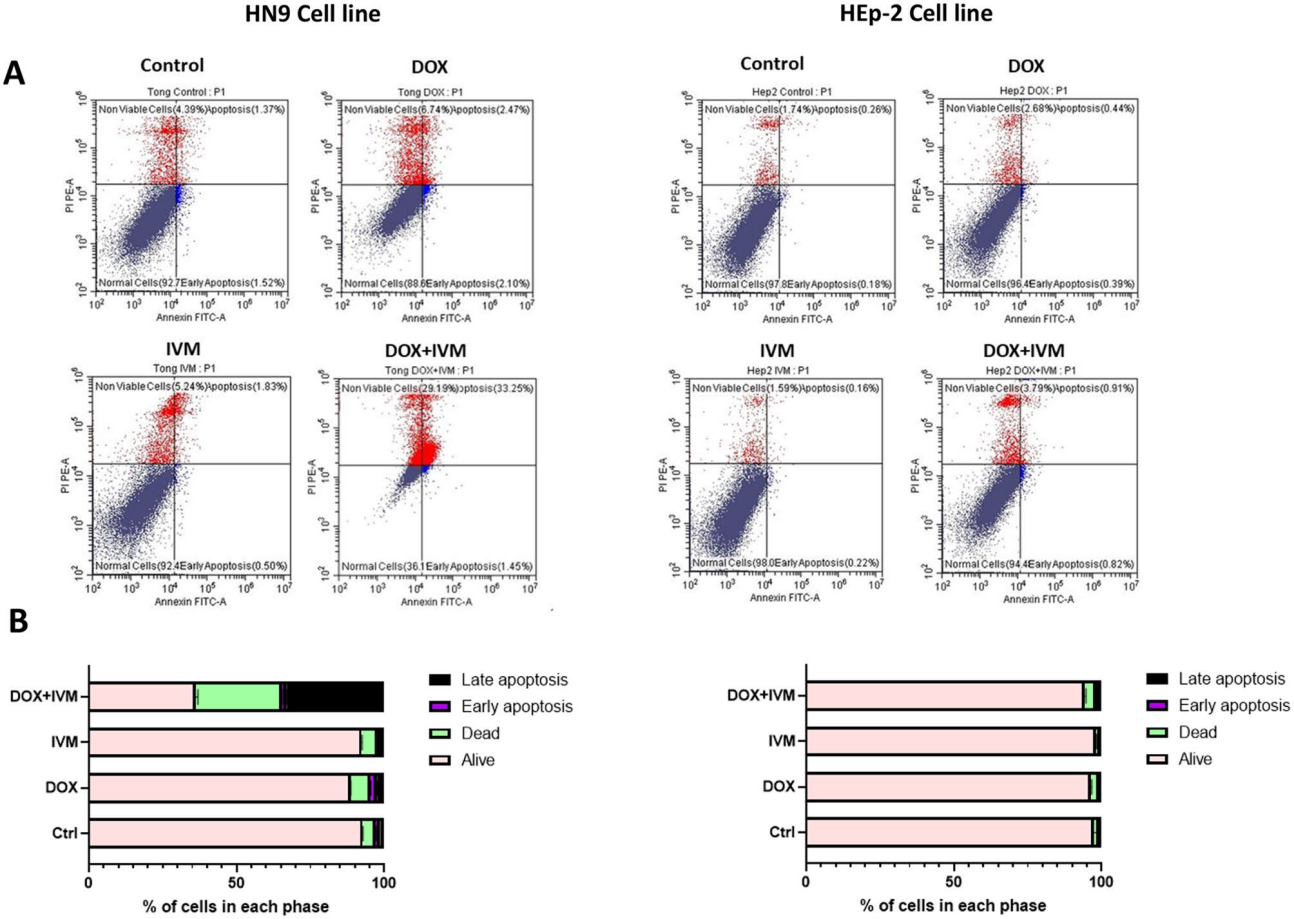
Apoptosis assay via Annexin V/PI demonstrating the percentage of apoptosis in HN9 and HEp-2 cell lines after treatment with DOX, IVM, and DOX + IVM combination. **(A)** Dot plots (Annexin V-FITC versus PI) presenting the population of living cells, early apoptotic cells, late apoptotic cells, and dead cells following exposure to DOX, IVM, and DOX + IVM for 48 h. **(B)** Bar chart illustrating the proportion of cells across each phase. Data are expressed as mean ± SD. One-way ANOVA and Tukey’s post-hoc statistical analyses were employed

Annexin V/PI staining was conducted to assess the apoptotic potential of DOX, IVM, and their combination in HN9 and HEp-2 cell lines (Fig. 2A). Statistical analysis revealed that IVM treatment significantly augmented the sensitivity and responsiveness of HN9 cells to DOX. The percentage of viable cells decreased significantly when cells were treated with DOX + IVM drug combination (36.23 ± 0.67) compared to the control (92.73 ± 0.13), DOX individually (88.67 ± 0.05), and IVM alone (92.42 ± 0.10) (*p* < 0.0001). In contrast, the percentage of cells in the late apoptotic phase and the percentage of dead cells were significantly increased by using the DOX + IVM combination (*p* < 0.0001) (Fig. 2B). In comparison, HEp-2 cells were more resistant to the DOX + IVM combination, maintaining higher cell viability (94.47% ± 0.19). However, there was a slight reduction in the percentage of viable cells in the HEp-2 cell line by 2.8% compared to the control.

### Cell cycle analysis

**Fig. 3:**
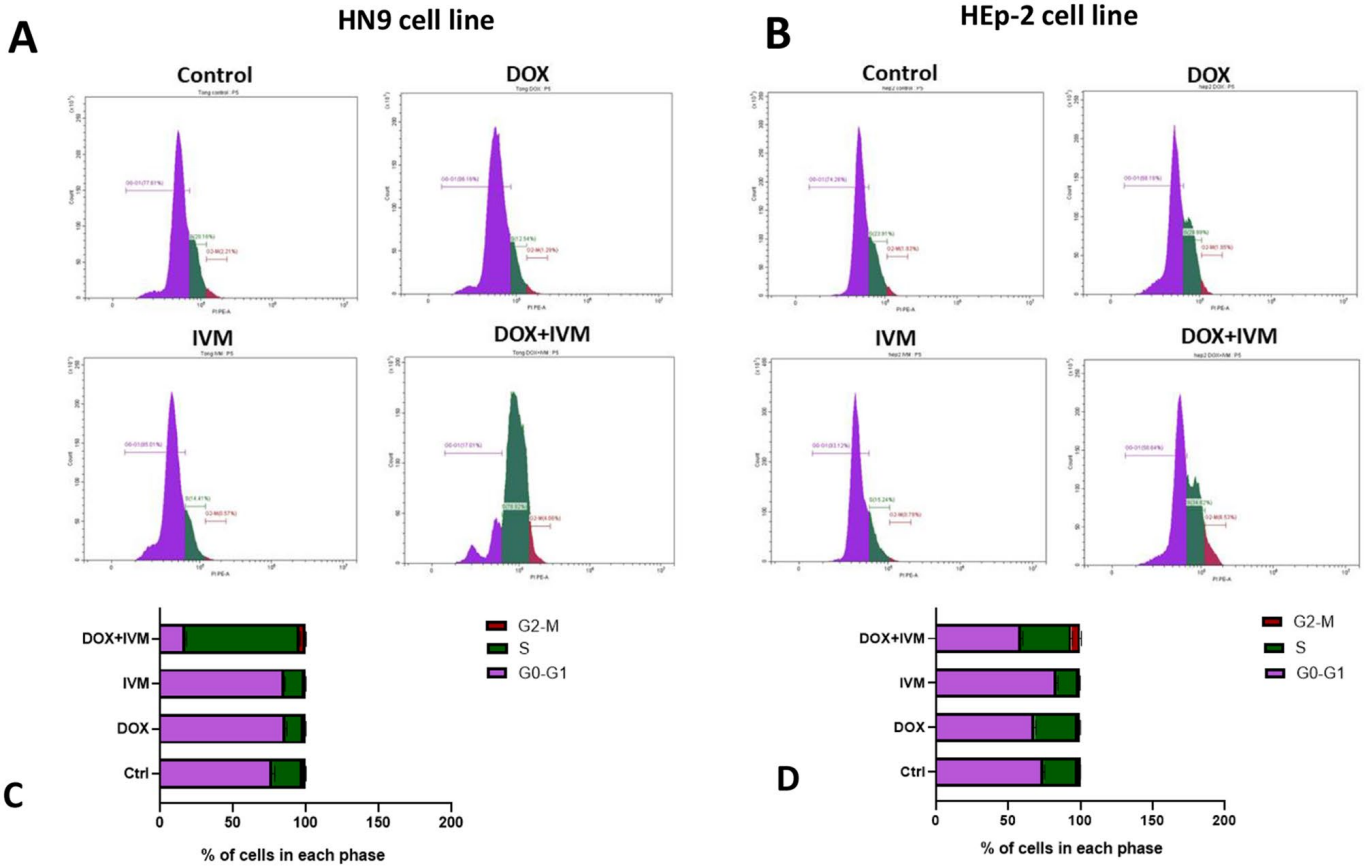
Analysis of cell cycle progression in HN9 and HEp-2 cell lines after treatment with DOX, IVM, and DOX + IVM combination for 48 h. (**A**-**B**) Histograms showing the distribution of cell populations across different cell cycle phases. (**C**-**D**) Bar chart showing the percentage of cells in each phase of the cell cycle. DOX + IVM combination exhibited cell cycle arrest at the S phase in HN9 and HEp-2 cell lines. Findings are revealed as mean ± SD. Data analysis was conducted via One-way ANOVA, then Tukey’s post-hoc test

Cell cycle analysis by flow cytometry was performed to evaluate the distribution of cells in each phase of the cell cycle after treating the cells with the previously determined concentrations. In HN9 cells, the results revealed that DOX and IVM individually didn’t cause significant cell cycle arrest at the S phase with the used concentrations; however, the combination exhibited the most significant arrest (by 4-fold) at the S phase relative to the control, DOX, and IVM individually (*p* < 0.0001, Fig. 3A-C)., 3B, D).

### The expression of apoptosis/ proliferation and Proinflammatory markers

**Fig. 4:**
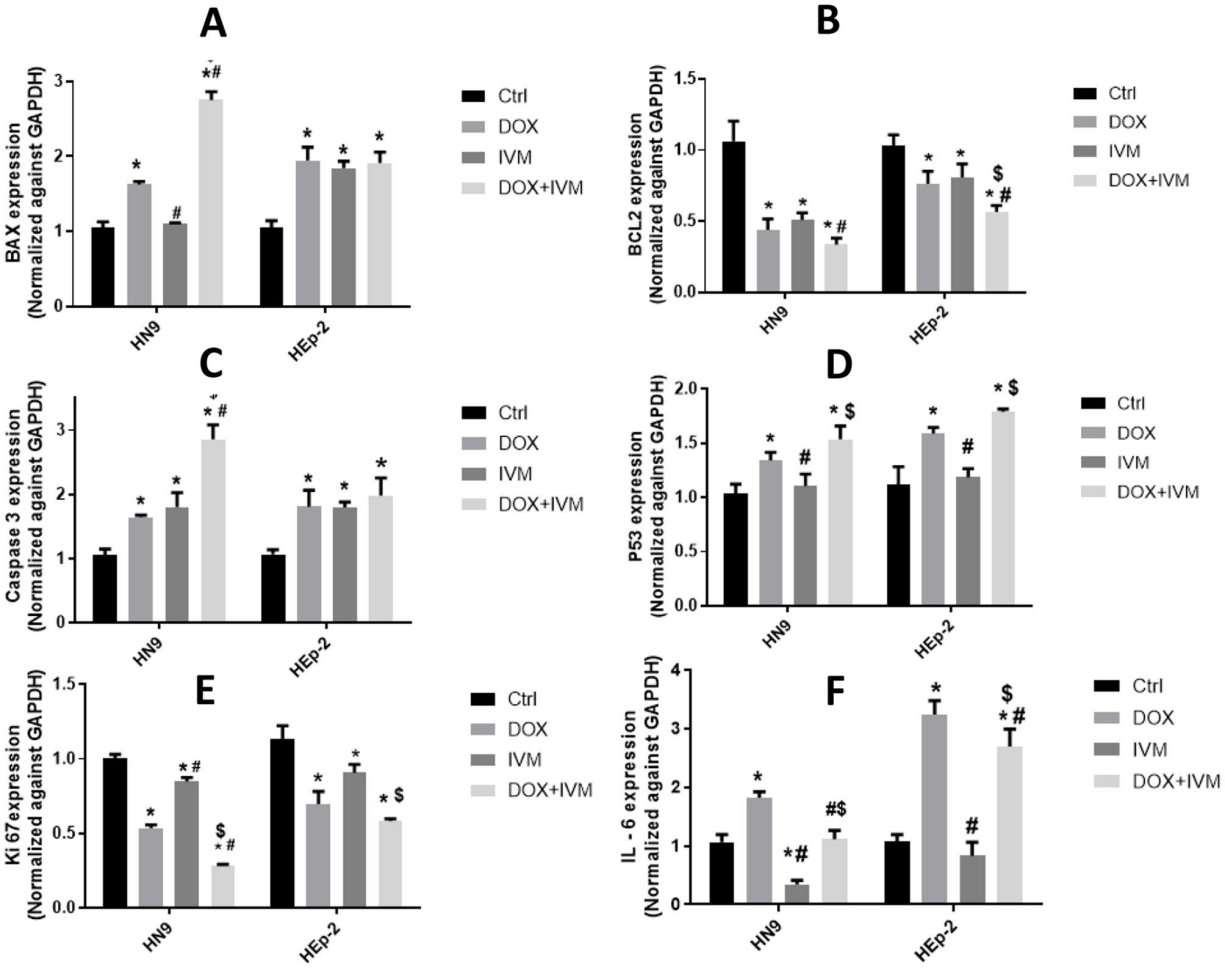
Charts presenting the fold change in mRNA expression of apoptotic proteins (BAX, BCL2, Caspase 3, and P53), proliferation marker (Ki-67), and proinflammatory marker (IL-6), assessed using RT-qPCR and calculated using the 2^−ΔΔCT^ method, of HN9 and HEp-2 cells subsequent exposure to DOX, IVM their combination. **(A)** BAX, **(B)** BCL-2, **(C)** Caspase-3, **(D)** P53, **(E)** Ki-67, **(F)** IL-6. Outcomes are revealed as mean ± SD. Statistical analysis was calculated using two-way ANOVA followed by Tukey’s *post-hoc* test. *p denotes pronounced difference between the respective group in relation to the control group, p denotes pronounced difference between the respective group in relation to the DOX group, ^**$**^p denotes pronounced difference between the respective group in relation to the IVM group. All samples were evaluated in triplicate (*n* = 3), and the experiment was repeated three times

Apoptosis-related markers (BAX, Caspase 3, and P53) were evaluated in HN9 and HEp-2 cell lines following treatment with DOX and IVM, individually and in combination. In the HN9 cell line, cells exposed to DOX showed higher expression of BAX relative to the cells subjected to IVM. Whereas cells treated with the DOX + IVM combination showed the most significant increase in BAX expression, with a 2-fold increase compared to the control group (*p* < 0.0001). In the HEp-2 cell line, exposed cells to DOX and IVM individually and in combination showed higher expression of BAX than the control group (*P* < 0.0001). However, no significant difference was observed between the DOX, IVM groups, and their combination (Fig. 4A).

In contrast, the results revealed a significant reduction in the expression of the anti-apoptotic marker BCL2 in the DOX- and IVM-treated groups, individually and in combination, in the HN9 and HEp-2 cell lines. The most significant reduction in BCL2 expression was observed in the DOX + IVM group, which showed a three-fold decrease in BCL2 expression relative to the control group in HN9 cells (Fig. 4B).

Caspase 3 expression was assessed in HN9 cells. Results showed that (Fig. 4C) the DOX + IVM group exhibited the most significant increase in Caspase 3 expression, compared to DOX and IVM individually. In comparison to the control group, the combination group showed a two-fold higher expression (*P* < 0.0001) of caspase 3. Moreover, no significant difference was noticed between the groups treated with DOX or IVM individually. In the HEp-2 cell line, cells treated with DOX, IVM individually, and in combination showed comparable levels of caspase 3 expression, indicating a more significant effect of the DOX + IVM combination on the HN9 cell line.

P53 expression in both cell lines showed an increased level in the DOX-treated groups compared to the IVM-treated groups (Fig. 4D). The DOX + IVM group exhibited the most significant increased expression of P53 compared to the IVM and control groups (*P* < 0.0001).

The proliferation marker expression, Ki-67, showed a significant reduction in DOX-treated cells compared to IVM in both cell lines. The DOX + IVM-treated cells revealed the most significant reduction compared to the DOX IVM-treated cells in the HN9 cell line (Fig. 4E). The expression of Ki-67 was reduced threefold in the combination treatment group relative to the control group. Similarly, in the HEp-2 cell line, the DOX-treated cells showed a significant reduction in Ki-67 expression compared to the IVM-treated cells, while the DOX + IVM-treated cells showed the least expression compared to the control group and each drug individually.

Interleukin 6 (IL-6) expression was markedly elevated (*P* < 0.0001) after treating the cells in both cell lines with DOX alone, denoting the triggering of the inflammatory pathway by the drug. In contrast, IL-6 expression was reduced significantly in the IVM-treated cells. Interestingly, IVM in the DOX + IVM combination groups had markedly reduced the IL-6 expression that was significantly elevated by the action of DOX. (Fig. 4F).

### Oxidative stress

**Fig. 5:**
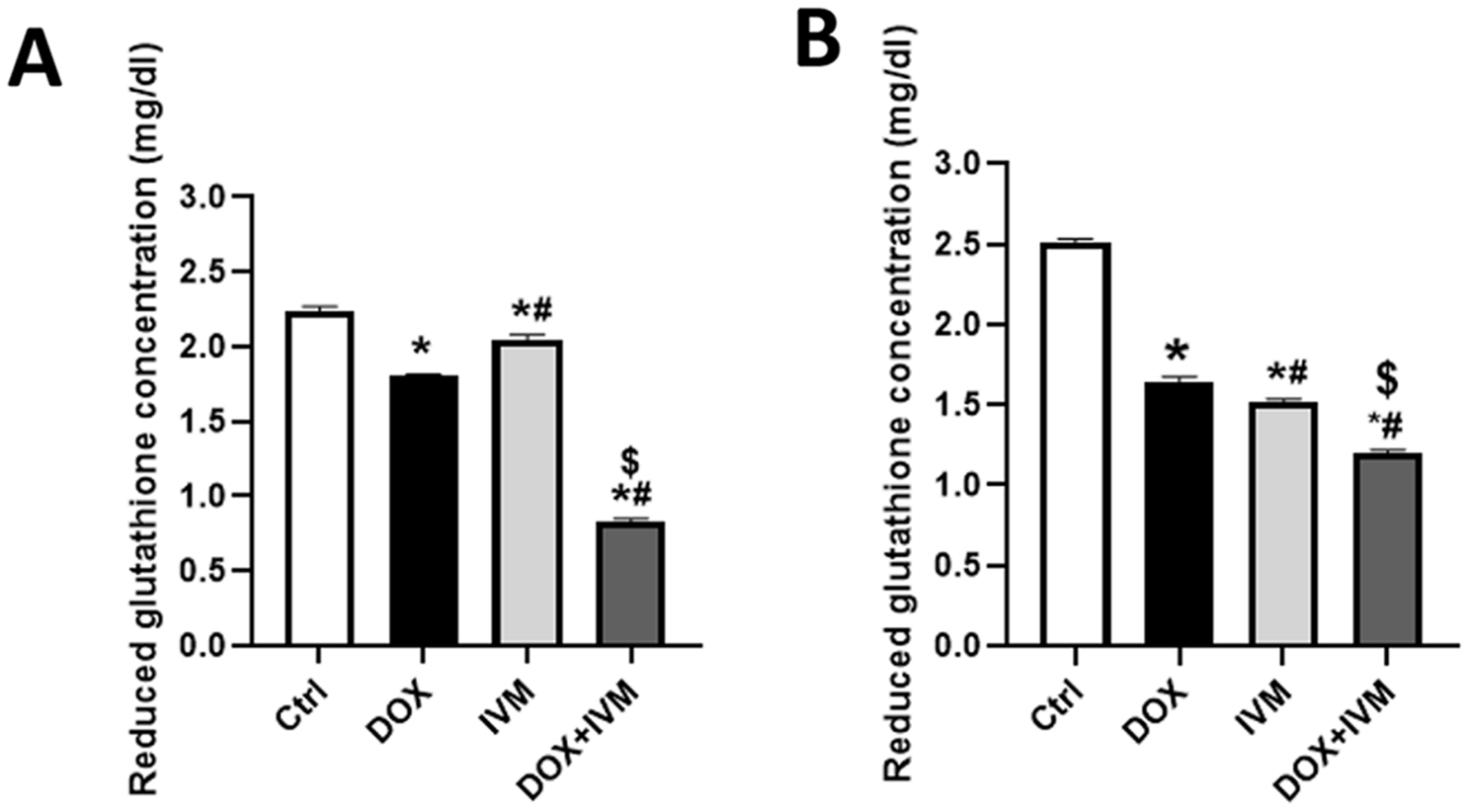
GSH levels in HN9 and HEp-2 cells after treatment with DOX, IVM, and DOX + IVM **(A)** The concentration of GSH in HN9 cells **(B)** The concentration of GSH in the HEp-2 cell line. The obtained data are presented as mean ± SD. Statistical analysis was employed via One-way ANOVA, then Tukey’s post-hoc test. *p denotes the marked difference between the respective group in comparison with the control group, p denotes the marked difference between the respective group compared to the DOX group, and ^**$**^p denotes the significant difference between the respective group relative to the IVM group

The results (Fig. 5A) denoted that the DOX + IVM combination significantly reduced the concentration of GSH, about a 2-fold reduction, compared to the control, DOX, and IVM groups in the HN9 cell line (*p* < 0.0001). This indicates an increase in oxidative stress using the combination.

Similarly, the concentration of GSH was more significantly reduced in the HEp-2 cell line following treatment with the DOX + IVM combination compared to the control, DOX, and IVM groups. (Fig. 5B). DOX alone decreased the concentration of GSH in both cell lines significantly compared to the control and IVM groups. In both cell lines, IVM enhanced the effect of DOX in the combination group, leading to a more pronounced reduction in GSH levels compared to each drug alone; however, the reduction in GSH level was more significant in the HN9 cell line compared to the HEp-2 cell line using the combination.

## Discussion

Oral squamous cell carcinoma (OSCC) remains a major global health challenge due to its aggressive behavior, limited survival rates, and resistance to conventional chemotherapy [3, 8, 15]. Although DOX is an established chemotherapeutic drug, its clinical use in OSCC is limited by chemoresistance, cardiotoxicity, and a narrow therapeutic index [12, 27]. Therefore, repurposing agents that can enhance DOX efficacy while reducing its adverse effects represents a promising approach [12].

IVM is a promising anticancer agent that selectively targets cancer cells while sparing normal cells and modulates multiple oncogenic signaling pathways, as evidenced by many in vitro and in vivo studies [17, 28]. However, the anti-cancer effectiveness of IVM and the combined effect of DOX + IVM in oral cancer have not yet been researched.

In this study, we examined the anticancer efficacy of the DOX + IVM combination in oral cancer cells. To ensure the reliability of our findings, all experiments were repeated using the HEp-2 cell line. Our results were validated through a series of in vitro assays, including MTT assay, cell apoptosis analysis, and cell cycle evaluation. Additionally, we evaluated apoptotic, proinflammatory, and proliferation markers using RT-qPCR. Finally, we investigated the effects of the drugs on oxidative stress.

The MTT assay was employed to assess the effects of IVM and DOX on the viability of OEC HN9 and Hep-2 cells. IC_20_ of DOX combined with a safe dose of IVM that maintained >90% cell viability was used to maintain an adequate number of cells for further molecular analysis. The results indicated that the combination of DOX and IVM exhibited the highest cytotoxic effects on both cell lines when compared to each drug alone. Furthermore, the DOX + IVM combination showed strong synergism (CI < 0.8), consistent with prior evidence that IVM enhances the activity of conventional chemotherapeutics by modulating apoptosis and oxidative stress pathways [29–31].

Mechanistically, the DOX IVM combination caused pronounced apoptosis and S-phase cell cycle arrest in both HN9, and HEp-2 cell lines. The S phase is the most crucial in the cell cycle, during which DNA replication and repair occur; thus, many chemotherapeutic drugs exert their cytotoxic effects on cells by targeting this phase [31]. DOX was previously reported to induce apoptosis through DNA intercalation and topoisomerase II inhibition, which ultimately resulted in DNA and cell membrane damage [32].

Another study by Zhou et al. reported that IVM increased the percentage of apoptotic colorectal cancer cells and caused cell cycle arrest at the S phase [29]. In contrast, IVM combined with gemcitabine induced cell cycle arrest at the G1 phase when used on pancreatic cancer cells [28].

The expression of apoptosis-related proteins (BAX, Caspase 3, and P53), along with the anti-apoptotic protein BCL2, was assessed in HN9 and HEp-2 cell lines. The results demonstrated that the DOX + IVM combination induced the most significant upregulation of BAX, Caspase 3, and P53 expression, while concurrently exhibiting the most significant reduction in BCL2 expression in both cell lines. Apoptosis can be initiated through two distinct pathways: intrinsic and extrinsic. An increased BAX/BCL2 ratio triggers apoptosis by inducing permeability of the mitochondrial membrane, which leads to the release of cytochrome c, triggering cascades of caspases, and finally results in programmed cell death [29, 30]. Our findings are consistent with those of Zhang et al. (2019), who demonstrated that HELA cells exposed to IVM resulted in a pronounced upregulation of Caspase 3 and P53 expression levels [33]. Moreover, IVM has been shown to induce apoptosis in glioblastoma cells [34] and human urothelial carcinoma cells [23] through a caspase-dependent pathway.

Ki-67 protein is a cell proliferation marker, used for grading multiple types of human cancers [35], its elevation is correlated with disease progression and poor prognosis in OSCC [36]. The present study revealed that the DOX + IVM combination caused the most significant decrease in Ki-67 expression in both cell lines. Our results are in accordance with those of Lee et al. (2022), who reported that IVM inhibited the proliferation of pancreatic cancer cells via Akt/mTOR phosphorylation [28]. IL-6 is a pro-inflammatory cytokine that plays a pronounced role in cancer metastasis and is clinically associated with poor prognosis in cancer patients. Moreover, IL-6 is a cytokine that is abundantly present in the tumor microenvironment of various tumor types, including HNSCC [37]. Our results showed that IL-6 was markedly elevated in cells treated with DOX alone, likely due to ROS production and subsequent oxidative stress mediated by DOX [38]. In contrast, IVM alone reduced IL-6 expression, and the DOX + IVM combination showed the most significant reduction in IL-6 expression, suggesting that IVM not only potentiates the cytotoxic effects of DOX but also mitigates its associated inflammatory response, which contributes to one of its most critical adverse effects, cardiotoxicity. To the best of our knowledge, there has been no previous report on the effects of Ki-67 and IL-6 expression in cancer cells treated with IVM, which highlights the potential role of IVM in suppressing tumor cell proliferation. However, the anti-inflammatory properties of IVM have been documented in infectious models [39].

To further elucidate the mechanism of the synergistic effect between DOX and IVM, the intracellular reduced glutathione (GSH) concentration was measured as an indicator of oxidative stress. Oxidative stress, marked by excessive reactive oxygen species (ROS) production [40], reduces mitochondrial membrane potential, releases cytochrome c, activates caspases, and triggers intrinsic apoptosis [17]. In the current study, it was demonstrated that IVM had a significant effect on cancer cells’ oxidative stress levels, with the highest increase in either cell line occurring after the DOX-IVM combination. Our findings are consistent with previous studies reporting that IVM induces apoptosis by promoting ROS generation, decreases mitochondrial membrane potential, as observed in colorectal cancer cells [29], renal cell carcinoma, glioblastoma, leukemia [17], and HELA cells [33]. This is aligned with previous studies demonstrating that IVM effectively potentiated the action of DOX in osteosarcoma cells [41] and enhanced the cytotoxicity of gemcitabine in pancreatic cancer cells [28] by inducing apoptosis through oxidative stress, which leads to mitochondrial dysfunction.

Despite these promising results, it is important to acknowledge certain limitations. The study was conducted exclusively in vitro, which does not fully replicate the complexity of the tumor microenvironment. Additionally, in the flow cytometry experiments, the unstained panel is preferred to be clearly annotated to show gating strategies. Furthermore, OSCC is genetically and phenotypically heterogeneous, and responses may vary across different subtypes. Therefore, future studies should include in vivo models to assess therapeutic efficacy, pharmacokinetic profiling, and molecular docking to identify synergistic mechanisms. Clinical studies will ultimately be necessary to determine translational relevance.

## Conclusion

The present study suggests that IVM enhances the anticancer activity of DOX in OSCC by promoting apoptosis, suppressing proliferation and inflammation, and inducing oxidative stress. This synergistic interaction positions IVM as a potential chemosensitizer in OSCC therapy, offering a promising avenue for improving treatment outcomes while reducing the toxic burden of high-dose DOX.

## Supplementary Information

Below is the link to the electronic supplementary material.

Supplementary Material 1

Supplementary Material 2

## Data Availability

Not applicable.
