# Supplementary material for: Synergistic potential of Ivermectin and doxorubicin in oral squamous cell carcinoma: an in vitro investigation
Source: BMC Pharmacol Toxicol. 2025 Dec 12;27:16. doi: 10.1186/s40360-025-01053-4 (PMC12817848; doi:10.1186/s40360-025-01053-4)
Supplement: Supplementary file 1 — Supplementary Material 1 [file 40360_2025_1053_MOESM1_ESM.docx]

**Supplementary Data A**

MTT assay results using a serial dilution of IVM ( 30 μM -0.23 μM) combined with the IC_20_ value of DOX showed that the IC_50_ of DOX+IVM was 9 uM in normal OEC, while was 1.79 uM in HN9 cell lines. Findings revealed a 4.5- fold elevation in the IC_50_ of DOX+IVM in cancer cells compared to normal cells . The IC_50_ of DOX+IVM in OEC cell line showed >80% cell viability, which is considered relatively safe according to the ISO 10993-5:2009 guidelines (1). Thus, it was determined to perform the following assays excluselively in cancer cell lines.


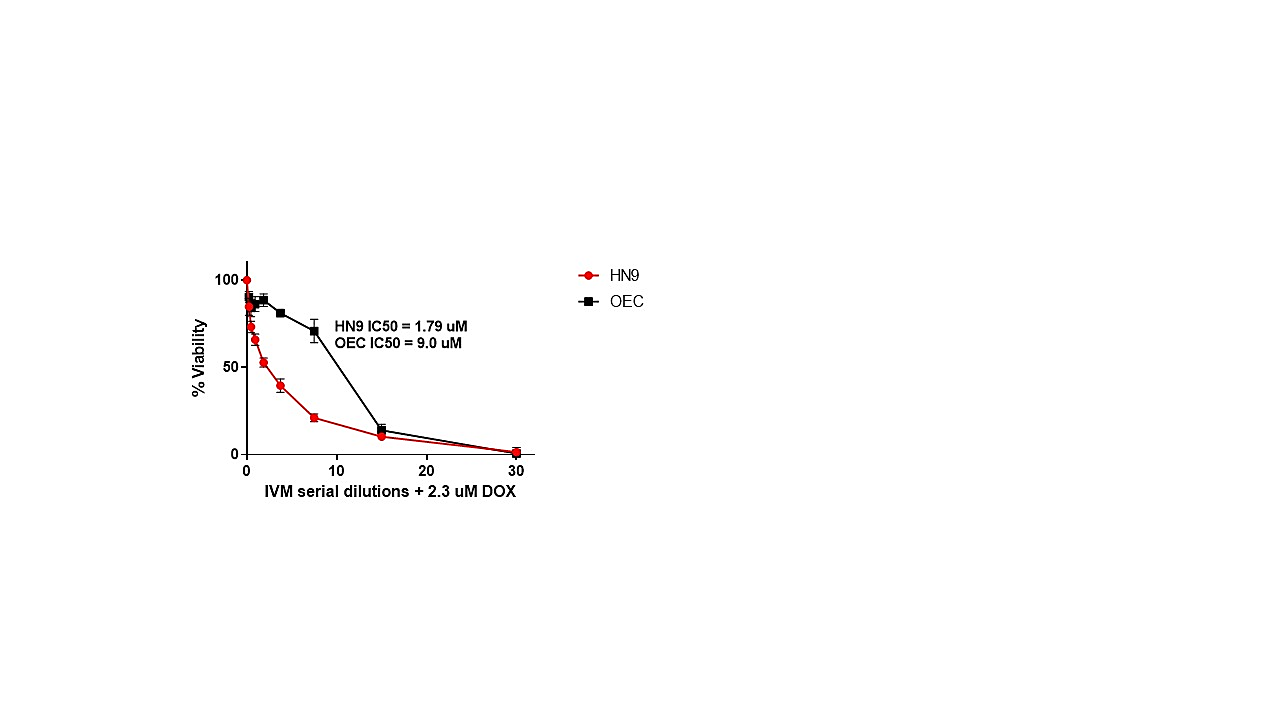


**Supplementary material 1.** MTT assay showing results of the effect of using a serial dilution of IVM ( 30 μM -0.23 μM) combined with The IC**_20_** value of DOX on OEC and HN9 cell lines.

Reference 1:

Gruber, S., & Nickel, A. (2023). Toxic or not toxic? The specifications of the standard ISO 10993-5 are not explicit enough to yield comparable results in the cytotoxicity assessment of an identical medical device. *Frontiers in Medical Technology*, *5*, 1195529. https://doi.org/10.3389/FMEDT.2023.1195529
