# Supplementary material for: Synergistic potential of Ivermectin and doxorubicin in oral squamous cell carcinoma: an in vitro investigation
Source: BMC Pharmacol Toxicol. 2025 Dec 12;27:16. doi: 10.1186/s40360-025-01053-4 (PMC12817848; doi:10.1186/s40360-025-01053-4)
Supplement: Supplementary file 2 — Supplementary Material 2 [file 40360_2025_1053_MOESM2_ESM.docx]

**Supplementary Data B**

**A**


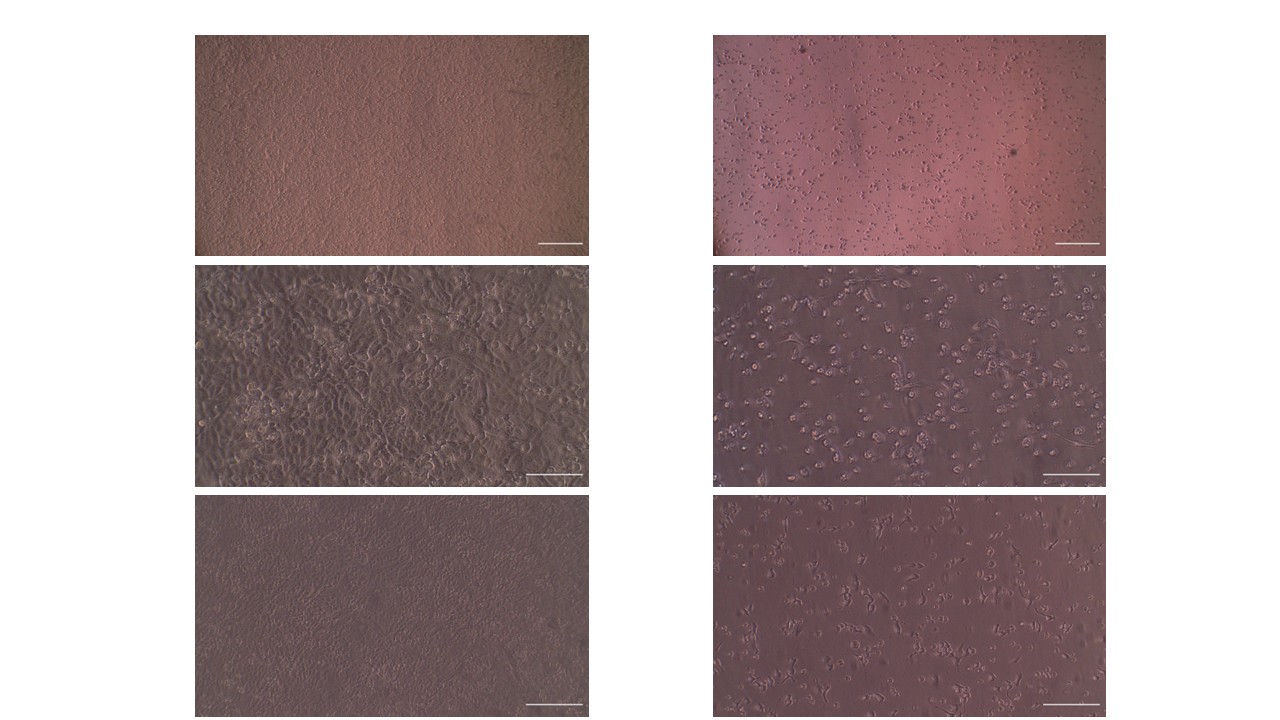


**10x**

**20x**

**4x**

**B**

**Representative microscopic images of HN9 cells before and after treatment with DOX+IVM**. (A) Control cells exhibited normal morphology with intact membranes. (B) DOX+IVM treated cells for 48 h showed marked morphological alterations such as cell rounding, shrinkage, and detachment. Images were obtained under inverted microscope at 10x, 20x, and 4x magnification (*Scale bar* =250, 125, 500 μm, respectively).
